# Countrywide Mortality Surveillance for Action in Mozambique: Results from a National Sample-Based Vital Statistics System for Mortality and Cause of Death

**DOI:** 10.4269/ajtmh.22-0367

**Published:** 2023-04-10

**Authors:** Ivalda Macicame, Almamy M. Kante, Emily Wilson, Brian Gilbert, Alain Koffi, Sheila Nhachungue, Celso Monjane, Pedro Duce, Antonio Adriano, Sergio Chicumbe, Ilesh Jani, Henry D. Kalter, Abhirup Datta, Scott Zeger, Robert E. Black, Eduardo Samo Gudo, Agbessi Amouzou, the COMSA-Mozambique study team

**Affiliations:** 1 Instituto Nacional de Saude, Maputo, Mozambique;; 2 Johns Hopkins Bloomberg School of Public Health, Baltimore, Maryland;; 3 Instituto Nacional de Estatistica, Maputo Mozambique

## Abstract

Sub-Saharan Africa lacks timely, reliable, and accurate national data on mortality and causes of death (CODs). In 2018 Mozambique launched a sample registration system (Countrywide Mortality Surveillance for Action [COMSA]-Mozambique), which collects continuous birth, death, and COD data from 700 randomly selected clusters, a nationally representative population of 828,663 persons. Verbal and social autopsy interviews are conducted for COD determination. We analyzed data collected in 2019–2020 to report mortality rates and cause-specific fractions. Cause-specific results were generated using computer-coded verbal autopsy (CCVA) algorithms for deaths among those age 5 years and older. For under-five deaths, the accuracy of CCVA results was increased through calibration with data from minimally invasive tissue sampling. Neonatal and under-five mortality rates were, respectively, 23 (95% CI: 18–28) and 80 (95% CI: 69–91) deaths per 1,000 live births. Mortality rates per 1,000 were 18 (95% CI: 14–21) among age 5–14 years, 26 (95% CI: 20–31) among age 15–24 years, 258 (95% CI: 230–287) among age 25–59 years, and 531 (95% CI: 490–572) among age 60+ years. Urban areas had lower mortality rates than rural areas among children under 15 but not among adults. Deaths due to infections were substantial across all ages. Other predominant causes by age group were prematurity and intrapartum-related events among neonates; diarrhea, malaria, and lower respiratory infections among children 1–59 months; injury, malaria, and diarrhea among children 5–14 years; HIV, injury, and cancer among those age 15–59 years; and cancer and cardiovascular disease at age 60+ years. The COMSA-Mozambique platform offers a rich and unique system for mortality and COD determination and monitoring and an opportunity to build a comprehensive surveillance system.

## INTRODUCTION

Low- and middle-income countries (LMICs), especially those in the sub-Saharan Africa region, lack timely data on mortality and causes of death (CODs). No country in the region has a national data system that generates reliable annual national statistics on mortality and COD for all ages. The heavy reliance by these countries on periodic household surveys and censuses to produce mortality statistics has in part led to little investment in national systems for continuous mortality and COD data collection. There has been enormous progress in the collection and analysis of mortality data through household surveys.^1^ However, the mortality statistics generated are often outdated by the time they are published and, most importantly, are averages over several years in the past, making them less useful for accurate assessment of the recent impact of national health programs. In addition, few national surveys incorporate modules for COD assessment through verbal autopsies (VAs). All African countries have established civil registration and vital statistics systems (CRVSs) that aim for complete recording of vital events, including births, deaths, and COD. However, such systems are defective, and, despite recent efforts for strengthening, achieving universal completeness and quality will take decades.^2^^,^^3^ Health and demographic surveillance sites also collect data on vital events, but their usefulness is constrained by their limited geographic scope making them nonrepresentative of the national situation.^4^^–^^6^

For LMICs, a promising approach that combines sample household surveys with demographic surveillance is the sample vital registration system with COD (SVRS).^7^^–^^9^ The idea of SVRS is not new, yet the approach has not been widely implemented by LMICs. SVRS focuses on a sample of communities, randomly selected across the country, from which selected vital events such as pregnancies, births, deaths (including CODs), and migration are monitored. Such data allow the generation of nationally representative demographic indicators such as population changes, fertility, mortality, and CODs, effectively providing real-time results for progress monitoring, decision-making, and resource allocation. Although a SVRS can be linked to and strengthens the CRVS, it is not designed to fulfill the universality of a CRVS; nor does it aim to issue legal documents, as in the case of the CRVS. As such, the word “registration” in SVRS does not refer to legal registration of vital events and often creates confusion among various stakeholders. A more accurate designation would be a sample-based vital statistics system. A major advantage of a SVRS is the ability to mount the system and make it fully functional within a short period of time and continue to maintain it over time. It also provides a sample of sentinel sites on which additional data can be routinely collected for surveillance of other outcomes, such as disease prevalence and child immunization.^10^

To date, only a few LMICs are successfully implementing such a system and generating continuous data that are used to monitor progress in the country. Countries with long-standing SVRS include India, Bangladesh, and China.^11^^–^^13^ Vietnam and Indonesia have also initiated the approach recently.^14^^,^^15^ A subnational SVRS has been attempted in Tanzania but has not been sustained due to lack of further investment.^16^ Mozambique launched a national SVRS system in January 2017 to generate mortality rates and COD data for all ages to support the monitoring of the Sustainable Development Goals for health.^17^ The project was titled Countrywide Mortality Surveillance for Action (COMSA) and has been implemented jointly by the Mozambique Instituto Nacional de Saude (INS) and the Instituto Nacional de Estatistica (INE). COMSA was motivated by interest in linking nationally representative data on causes of child death to another project, Child Health and Mortality Prevention Surveillance (CHAMPS), implemented in one district, that uses a laboratory-based testing of minimally invasive tissue samples (MITS) to determine CODs in children under age 5 years.^18^ A COMSA project is also being implemented in Sierra Leone covering 678 randomly selected geographic census enumeration areas and a total population of 340,000.^19^

We present in this paper the main results achieved by the COMSA project 4 years after its launch. We describe the demographic structure of the sample population, all-cause mortality rates, and cause-specific fractions by age groups. We also present selected results of the social autopsy analysis based on the pathway to survival framework^20^ and describe other potential uses of the surveillance system.

## MATERIALS AND METHODS

### The COMSA project.

**Figure 1.**
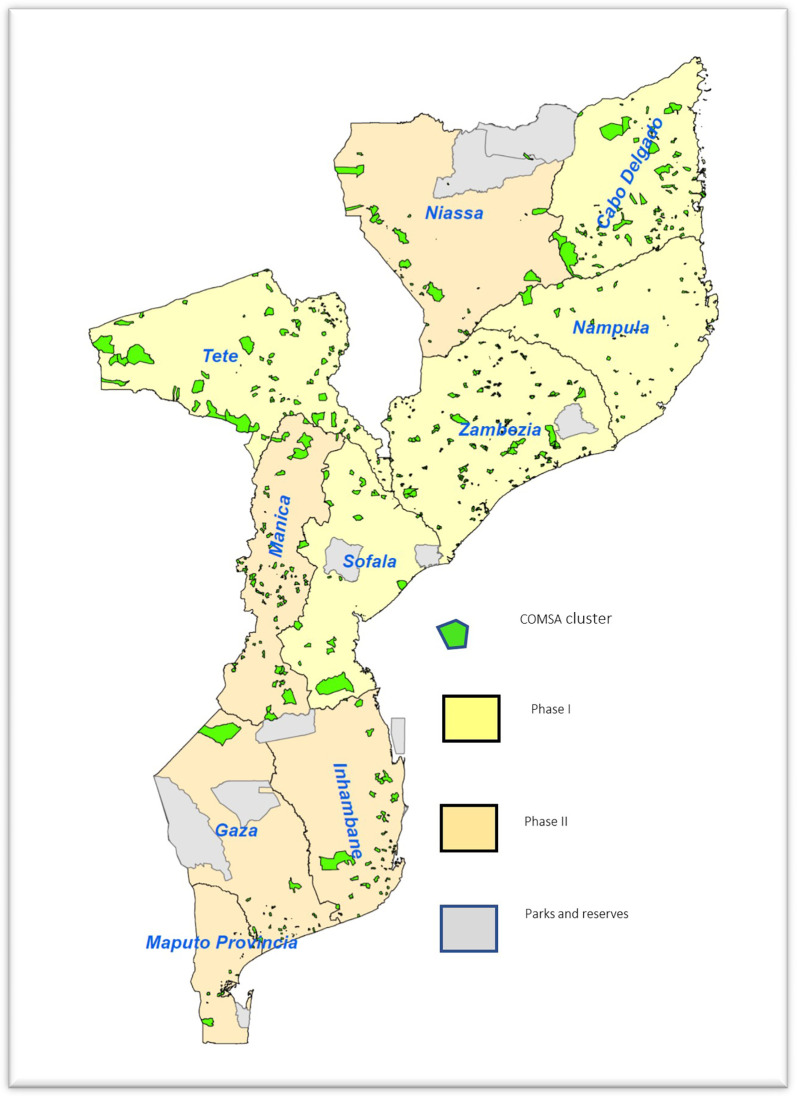
Map of Mozambique showing the distribution COMSA clusters in phase I and II provinces.

COMSA is based on routine community surveillance of pregnancy outcomes and deaths, followed by an annual census to update the population and assess completeness and quality of the surveillance resulting in a dual mortality recording system. The COMSA project is based on a representative, provincially stratified random sample of 700 geographic clusters. The clusters were defined based on the 2007 population census of control areas, which consisted of a group of one to three enumeration areas with approximately 300 households supervised by a census controller. The clusters were selected with probability proportional to population size (PPS). The sample size was calculated based on provincial estimates of infant mortality rate (IMR) using data from the 2011 Demographic and Health Survey (DHS).^21^ The IMR obtained from the 2011 survey was projected to 2016 using the United Nations estimate of annual rate of change between 2000 and 2010. We used relative error margins between 11% and 29% at the provincial level. Supplemental Appendix Table 1 presents the sample size and assumptions. Based on discussions with the Ministry of Health and the sponsor, four provinces with the highest under-five mortality, as estimated through the 2011 DHS, were oversampled to obtain sample sizes that are large enough to allow for annual monitoring of all-cause and cause-specific mortality rates with improved precision. These provinces were Cabo-Delgado, Manica, Tete, and Zambezia. Figure 1 presents the map of Mozambique showing the distribution of the COMSA clusters. Given the oversampling and the selection of clusters with PPS, we calculated sampling weights to adjust the national estimates.

The National Institute of Statistics officers visited each sample cluster to meet with community leaders and officials to identify and recruit a resident community surveillance agent (CSA). The CSAs were recruited, trained, and compensated specifically for the COMSA project. The CSAs, mostly residents of the clusters, were equipped with mobile phones loaded with a pre-programmed tool with open-data kit (ODK) data collection software to report prospectively data on pregnancies, pregnancy outcomes (live births and stillbirths), and deaths as they are identified. The CSA identified pregnancies that were physically visible or reported by the pregnant woman. The main objective of the pregnancy monitoring was to facilitate a follow-up to capture information on the birth outcome. The CSAs began their activities by performing a complete registration of all households within their clusters to establish the population by age and sex and obtain consent from heads of households for subsequent event monitoring. The CSAs continuously registered new households in the cluster; however, those who left were not systematically documented.

Another team of data collectors, composed of females with secondary or more schooling, were recruited and trained to conduct verbal and social autopsy (VASA) interviews. The choice of selecting only female data collectors was based on the initial formative study that revealed that, contextually, females were more effective and well-received at conducting interviews related to sensitive and emotional topics such as death. VASA data collectors were based at the provincial capital and visited all clusters monthly to conduct VASA interviews using tablets loaded with VASA tools programmed on ODK data collection software. Households with deaths were visited approximately 1 month after the mourning period has passed. The VASA tool integrates a social autopsy questionnaire with the 2016 WHO VA questionnaire.^22^^,^^23^ The VASA team also maintained continuous contact with the CSA and supervised them on their routine activities.

The COMSA project was launched in January 2017. However, actual data collection in five phase-one provinces started in March 2018. The remaining provinces, included in phase two, started data collection in October 2018. The annual updated census, planned in late 2019, was delayed first by the rainy season and subsequently by the COVID-19 pandemic. A provincially staggered census was completed in the second half of 2020, providing data to update the sample population and assess and complement birth events data reported in the years 2019 and 2020.

Due to the disruption caused by the COVID-19 pandemic in the 2020 data collection, the CSAs were further trained and deployed to collect data on births and deaths retrospectively for the year 2020. Thus, data reported here include a combination of the events data reported by the CSAs since the start of the project, updated with the census conducted in 2020, and the 2020 retrospective capture of events during the pandemic period.

### Methods of analysis.

#### Population age and sex structure.

For population structure, we used the updated population of COMSA clusters obtained during the 2020 census, adjusted for nonrespondent households (1.57%), and 40 clusters that were not surveyed due to security issues. We described the age structure of the population and compared it with the structure produced by the 2017 national population census.

#### Births, deaths, and mortality rates.

For the demographic rates and CODs, we report results for the period 2019–2020 after the community data had been merged with the 2020 assessment census data. We calculated the mortality rates for neonates (0–27 days), infants (0–11 months), and 5-year age groups. We also reported rates for wider age groups of 5–14, 15–24, 15–59; 25–59; 60+ years. For neonatal, infant, and under-five mortality, the rates were calculated by dividing the weighted number of deaths by the weighted number of live births for the same period. Under the assumption of slow changes in annual number of births, this conventional calculation is a good approximation of the true probability of death in this age group. For all other age groups, we first computed the death rate by dividing the weighted number of deaths in the age period by the population of the age period. These rates were then converted to probabilities using Equation (1).^24^

$${}_{n}q_{x}=\frac{n{*}_{n}M_{x}}{\left[1+(n-s){*}_{n}M_{x}\right]}$$ (1)

where *_n_q_x_* is the probability of death between ages *x* and *x* + *n*; *_n_M_x_* is the death rate between ages *x* and *x* + *n*; and *s* is the average number of person years lived in the interval by those who died in the interval *x*, *x* + *n*, generally taken as *n*/2 under the assumption of uniform distribution of deaths, for age groups above 5 years. We computed 95% confidence intervals (CIs) using a nonparametric jackknife method.

Using the distribution of probability of death by 5-year age groups and life table approach, we estimated life expectancy at birth at national level by sex, and by province. Estimates of crude birth rate (CBR) and crude death rate (CDR) were computed using the total population estimated from the 2020 census of clusters.

### CODs.

The COD analysis used VA data. At total of 5,930 deaths with VA were available for the period 2019–2020, with 3,401 in 2019 and 2,529 in 2020. There were 547 neonatal deaths and 1,194 deaths among those aged 1–59 months with VA information on this period. Different analytic methods were implemented for under-five children and older individuals due to the availability of auxiliary data for under-five children to calibrate the raw VA-based estimates. Deaths among under-five children were separated into neonatal and 1–59 months. For each group, we implemented two computer-coded VA (CCVA) algorithms, InsilicoVA and Expert Algorithm VA (EAVA), to generate the cause-specific mortality fractions (CSMFs) separately.^25^^,^^26^ The results were then calibrated using MITS-VA pairs data obtained from the CHAMPS project. The VA calibration method is detailed elsewhere.^27^^,^^28^ The procedure estimates the misclassification rates (error rates) of CCVA algorithms by cross-tabulating the VA-based and MITS-based CODs. These error rates are then used to improve the COMSA VA-based CSMF (a procedure referred to as VA calibration). The available CCVA methods often yield inconsistent results. Rather than relying on one method, we generated an ensemble calibrated CSMF using a weighted average of the method-specific CSMF from InSilicoVA and EAVA, with weights driven by the accuracy of these methods based on the MITS-VA error matrices. The MITS-VA pair data obtained from CHAMPS as of December 2020 included 340 neonates and 426 children 1–59 months; data were pooled across all the CHAMPS sites. The ensemble calibrated CSMFs are reported only at national level given limited sample size for a robust application at subnational levels. We report CODs by subregion (southern, central, and northern) and by place of residence using the InsilicoVA method. For older individuals 5–14, 15–59, and 60+ years old, we do not have MITS data to calibrate the VA results. We report the CSMFs using InsilicoVA.

### Social autopsy.

We analyzed social autopsy (SA) data of deaths of all ages in 2019–2020, focusing on the pathway to survival framework (Supplemental Appendix Figure 3). The framework describes careseeking patterns during the fatal illness or condition from home to the health facility and thus tries to identify failures on the careseeking pathway that could have contributed to the death. The steps start from illness recognition, decision and attempt to seek care outside the home, arrival and departure from the health provider, referral, and compliance with the referral. The analysis was stratified by the same age at death groups as the COD analysis.

All data analyses were carried out using Stata (version 16.1) and R (version 4.0.3) statistical software and the R package openVA (version 1.0.12).

### Ethical clearance.

The COMSA project has been approved by the National Health Bioethics Committee of Mozambique (REF 608/CNBS/17) and by the institutional review board of the Johns Hopkins Bloomberg School of Public Health (IRB#7867).

## RESULTS

### Population structure and age–sex composition.

**Figure 2.**
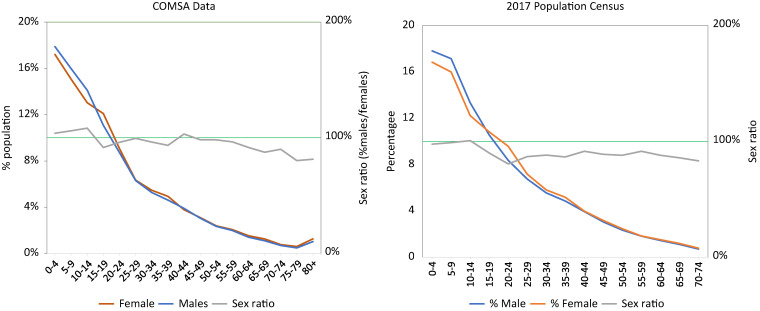
Distribution of male and female population and sex ratio comparing COMSA data and the 2017 population census.

The 660 geographic clusters that reported data throughout the period covered a population of 744,732 in 2020. When adjusted for the remaining 40 clusters, we estimated that the 700 clusters cover a population of 828,663. Like other countries with high fertility, the population is very young, with 18% under age 5 years and 47% under 15 years (Figure 2). The overall sex ratio is 94%, with an indication of a deficit of men between ages 15 and 40 years and over 65 years. The age structure and age–sex distribution of the COMSA population is comparable to the structure and distribution observed in the 2017 population census (Figure 2).

### Birth and death rates.

**Figure 3.**
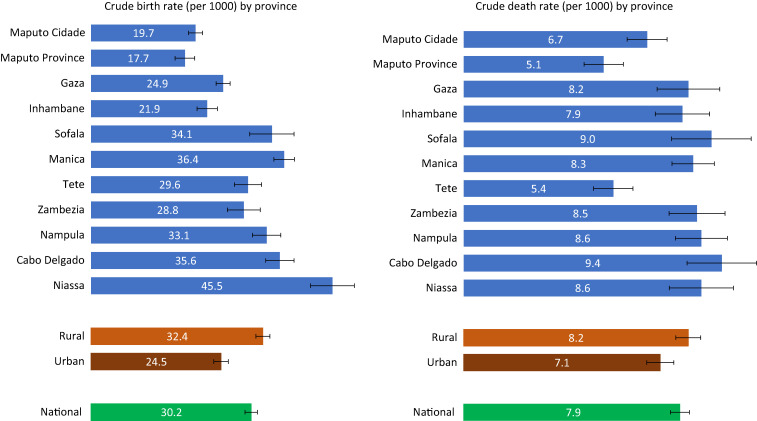
Crude birth and crude death rates by province, COMSA-Mozambique, 2019–2020.

We estimated a CBR and CDR, respectively, at 30.2 births (95% CI: 29.0–31.3) and 7.9 deaths (95% CI: 7.5–8.2) per 1,000 people in 2019–2020 (Figure 3). These rates were lower than those estimated by the 2017 population census estimates of 37.9 and 11.9, respectively. The provinces of Niassa and Cabo-Delgado in the north and Manica in the central region have the highest birth rates, and Maputo City and Maputo Province have the lowest rates. Birth rates were higher in rural area than urban by 8 per 1,000: 24.5 (95% CI: 23.1–26.0) versus 32.4 (95% CI: 31.1–34.0). The pattern for death rates does not mirror the birth rates. Provinces with the highest death rates were Cabo-Delgado and Sofala; Maputo City, Maputo Province, and Tete have the lowest death rates.

### Age-specific mortality rates.

**Figure 4.**
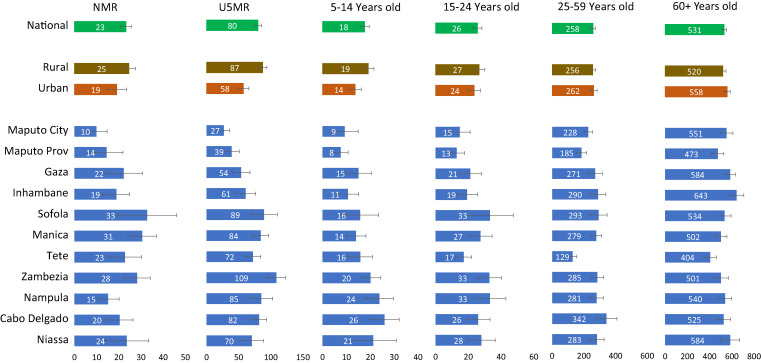
Neonatal mortality rate (NMR); under-five mortality rate (U5MR); 5–14, 15–24, 25–59, and 60+ years mortality rates, 2019–2020.

Mortality rates were generally high but variable across provinces and by urban or rural residence. We estimated neonatal and under-five mortality to be 23 (95% CI: 18–28) and 80 (95% CI: 69–91) deaths per 1,000 live births, respectively, in 2019–2020 (Figure 4). Under-five mortality ranged from 27 in Maputo to 109 in Zambezia province in the central region of the country. Urban areas have substantially lower neonatal and under-five mortality rates than rural areas, although the 95% CI between the two areas overlap. Neonatal mortality rate was estimated at 19 (95% CI: 10–28) in urban areas, compared with 25 (95% CI: 19–31) in rural areas; under-five mortality rates were 56 (95% CI: 41–75) in urban areas, compared with 87 (95% CI: 74–101) in rural areas

The mortality rate among children aged 5–14 years was 18 deaths per 1,000 at the national level (95% CI: 14–21). It was highest in the northern provinces (Cabo-Delgado, Niassa, Nampula, and Zambezia), reaching 26 in Nampula. It was lowest in Maputo City and Province, with 8–9 deaths per 1,000. Children aged 5–14 also faced higher risk of death in rural areas (19 deaths per 1,000; 95% CI: 14–24) than in urban areas (14 deaths per 1,000; 95% CI: 9–19).

The mortality rate among adolescents and young adults aged 15–24 years was estimated at 26 deaths per 1,000 (95% CI: 20–31), with large variability across provinces. Northern and central provinces, such as Zambezia, Nampula, and Sofala, had the highest rates. However, the difference between urban and rural areas was not statistically significant, with rates of 24 deaths per 1,000 (95% CI: 16–31) in urban areas, compared with 27 (95% CI: 20–34) in rural areas.

The mortality rates among adults 25–59 years old and 60+ years were 258 (95% CI: 229–287) and 531 (95% CI: 490–572) deaths per 1,000, respectively, in the years 2019–2020. Both rates varied across provinces, but only the mortality among individuals aged 60+ showed a substantial urban–rural difference, with urban areas indicating a higher risk of death than rural areas: 558 (95% CI: 504–612) versus 520 deaths per 1,000 (95% CI: 467–574).

Examination of reports of deaths by months and age group in 2019 and 2020 did not show any clear increase in deaths during the peaks of the COVID-19 pandemic in 2020 (Supplemental Appendix Figure 3). The estimates of mortality rates by 5-year age groups showed a J shape, with a high mortality rate in the extreme age groups (children and older adults) (Supplemental Appendix Figure 2). The distribution implied a life expectancy at birth of 63.2 years in 2019–2020, with a female advantage gap of 5.6 years over males (66.1 years versus 60.4 years; see Supplemental Appendix).

### Causes of neonatal and child death.

**Figure 5.**
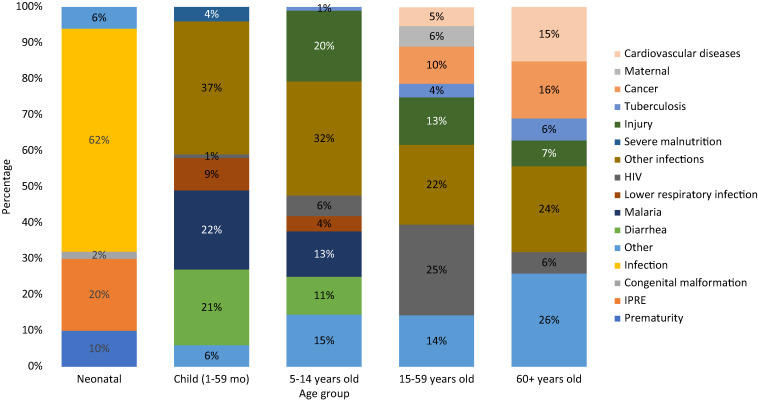
Cause of death fraction (%) by age group (COMSA data 2019–2020).

Calibrated VA results indicated that infections were the main COD among neonates and children in 2019–2020 in Mozambique, affecting 62% of neonates and 90% of children 1–59 months (Figure 5). Among newborns, conditions related to the quality of delivery care were substantial. Intrapartum-related events (IPREs, including birth asphyxia and birth trauma) and prematurity caused, respectively, 20% (95% CI: 14–26) and 10% (95% CI: 7–13) of deaths. Among children 1–59 months old, lower respiratory infections, HIV/AIDS, diarrhea, malaria, and tuberculosis were responsible for over half (53%) of child deaths. Other conditions included congenital malformations, cancer, sudden infant death syndrome, epilepsy, sickle cell disease, liver disease, heart disease, chronic skin ulcer, digestive disorders, injury, and others.

**Figure 6.**
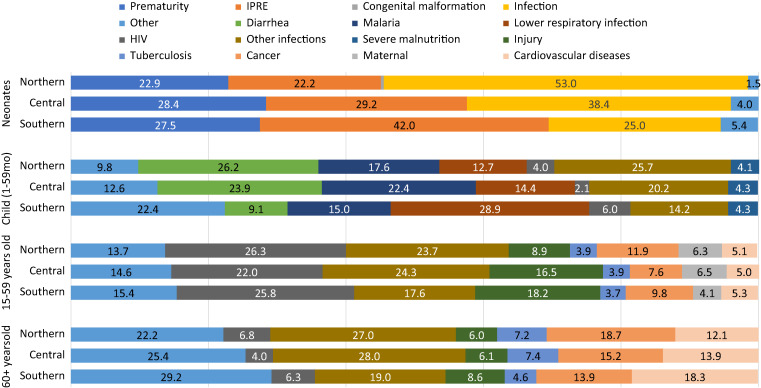
Cause of death fraction (%) by age group and region in Mozambique (COMSA data 2019–2020).

The distribution of CODs among neonates and children was similar across the three regions of the country, especially for child (1–59 months) causes (Figure 6). Among neonates, however, infection predominated in the northern and central provinces, whereas delivery conditions (IPRE) and prematurity were predominant in the southern region. Among children 1–59 months old, lower respiratory infection deaths were dominant in the southern region, whereas diarrhea and malaria deaths were more prevalent in the central and northern regions. Male neonates died more of IPRE than females whereas females died more of infections than males (Figure 6). Among children 1–59 months, the distribution of deaths by cause was generally similar between males and females, except for infections, which affected females more than males. These differences were, however, not statistically significant (Supplemental Appendix Table 5).

### CODs among children 5–14 years.

In children aged 5–14 years, injury was the top COD, contributing 20% (95% CI: 15–24) of deaths (Figure 4), followed by infections. Top infectious disease CODs included malaria (13%; 95% CI: 9–16) and diarrhea (11%; 95% CI: 7–14). The low number of deaths in this age group did not permit further disaggregation of the CODs by region. However, there were differences between male and female subjects (Figure 6). These differences were not statistically significant, although male children tended to die more of diarrhea and lower respiratory infections than female children.

### CODs among adults 15–59 and 60+ years.

AIDS was a major COD among those age 15–59 years (25%; 95% CI: 23–27). It was followed by other infections (22%; 95% CI: 20–24), including meningitis/encephalitis, dengue fever, etc. Injury was also a substantial COD, contributing 13% of deaths (95% CI: 12–15). This age group also faced a sizable burden of noncommunicable diseases, such as cancer and cardiovascular diseases, contributing to 10% (95% CI: 9–12) and 5% (95% CI: 4–6) of deaths, respectively. Maternal deaths contributed 6% (95% CI: 5–7) of all deaths in this age group. Infectious diseases caused 36% of deaths among seniors 60+ years. Noncommunicable diseases, such cancer and cardiovascular diseases, were prevalent, contributing, respectively, 16% (95% CI: 14–18) and 15% (95% CI: 13–17) of deaths. Figure 5 shows the distribution of COD by region.

**Figure 7.**
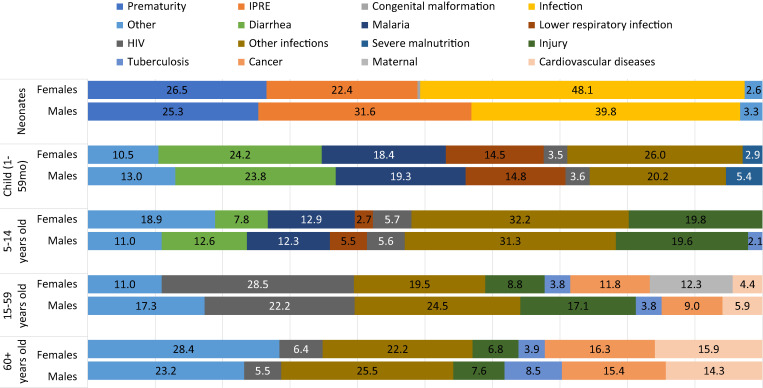
Cause of death fractions (%) by age group and sex in Mozambique (COMSA data 2019–2020).

The pattern of causes is similar across the three regions and by age groups. However, some substantial differences were noticeable by sex (Figure 7). Males aged 15–59 died more of injury and of other infections than females. Males also died more of HIV/AIDS than females, although the difference was not statistically significant. Among seniors aged 60+, a significant difference was observed for deaths due to tuberculosis, where men were twice as likely to die as women.

### Social autopsy: careseeking pathway to survival.

**Figure 8.**
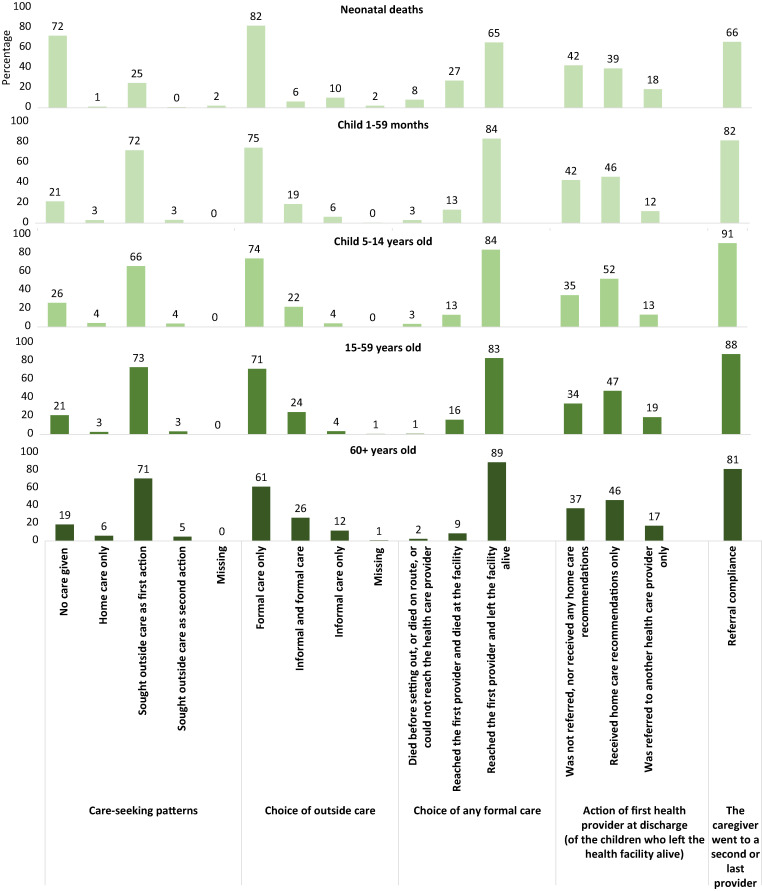
Indicators of the pathway for survival for neonates, children 1–59 months, children 5–14 years, and adults 15–49 and 50+ years old (COMSA data 2019–2020).

Figure 8 retraces the careseeking steps for neonates, children 1–59 months, children 5–14 years, and adults 15–59 years and 60+ years deaths during the fatal illness. The pattern differs mainly between neonatal deaths and other age groups, but in both cases multiple failures on the pathway to survival could be identified. Almost three-quarters (72%) of neonates died with no care. Care was sought outside the home as first action for only 25%, and 10% of those sought informal care only. Among those who sought formal care, delayed careseeking may have contributed to the death of 35%, either en route to the provider (8%) or after reaching the provider (27%). Although the majority of the neonates who reached the facility were later discharged alive (65%), about 40% of those were sent home without referral or any home care recommendation, 39% received only home care recommendations, and only 18% were referred. Compliance with referral faced challenges; only two-thirds (66%) of referred neonates complied with the referral. The pathway is similar for deaths of children and adults. About one in five received no care, whereas the majority (over 70%) sought care outside as a first action and generally sought formal care. Older individuals (50+ years) were more likely to seek informal care than younger individuals. Over 80% of those who sought formal care reached the provider and were discharged alive but without being referred. Children in particular were less likely to be referred compared with adults. Compliance with referral was high, at over 80%.

## DISCUSSION

Mozambique’s new SRVS, implemented under the COMSA project, is generating relevant health data required to assess progress on mortality reduction and programmatic targeting. The system covers 828,663 people in 700 clusters, randomly selected to generate nationally and subnationally representative mortality rates and CODs. Analysis of the data from 2019 to 2020 reveals levels of mortality rates comparable to the average rates in sub-Saharan Africa. The neonatal and under-five mortality estimates of 23 and 80 deaths per 1,000 live births, respectively, are close to modeled estimates (not including the COMSA data) from the United Nations Inter-agency Group for Child Mortality Estimation for 2020—29 (95% CI: 19–44) and 74 (95% CI: 48–107), respectively—with overlapping 95% CI. These rates were estimated at 27 and 74, respectively, for sub-Saharan Africa in 2020.^29^ Compared with estimated empirical mortality rates from the 2011 DHS, these rates indicate only a modest reduction in childhood mortality over the past decade.^30^ The 2011 DHS values for the neonatal and under-five mortality rates in the past decade were 30 and 97, respectively, which are 23% and 18% reductions. The mortality levels estimated by COMSA call for reinforcement of neonatal and child health programs to accelerate mortality reduction. This effort must target rural areas across all provinces, especially in northern and central provinces where child mortality was highest. We found that the urban area advantage over rural areas in child mortality disappeared among adults and reversed among seniors 60+ years. Provinces such as Zambezia, Manica, and Sofala experienced the highest neonatal and under-five mortality. The distribution of death rates by age shows highest rate at both extreme ages, leading to a life expectancy of 63.2 years, a rate slightly higher than estimated by the UN population division (61.2 years) in 2020 but that represents an improvement from the 2010 estimated life expectancy of 54.2 years.^31^ Like in all countries, females had higher life expectancy than males, due primarily to their lower death rates in older adults.

The COMSA-Mozambique project uses VA interviews with family members of the deceased to estimate the CODs. Given the large number of deaths, the VA analysis uses existing CCVA analysis algorithms to determine CODs. The VA approach has existed for decades and is accepted as a feasible alternative for determining COD in settings where most deaths occur in the community, where causes are not medically certified. CCVA methods have been validated against medically certified causes and are now more widely used.^21^^,^^24^^,^^32^^–^^34^ To improve COD estimation among neonates and young children, the COMSA project uses an innovative, recently proposed VA-calibration approach.^26^^,^^27^ The approach, first implemented with these data, requires accessing MITS results paired with VA data on a sample of deaths and using these data to generate the VA error (misclassification) matrix. The MITS data are collected by the CHAMPS project in one district and one city in Mozambique and in sites in seven other countries.^35^ The calibration is applied to each CCVA method (i.e., InsilicoVA and Expert Algorithm VA) for neonates and children 1–59 months old. The cause-specific mortality fractions for the two CCVA methods are combined in an ensemble calibrated result that is shown to be superior to each individual CCVA method.^26^ The findings indicate a large preponderance of infectious diseases as COD in these age groups. In neonates, these were followed by intrapartum-related events (birth asphyxia or birth injury) or complications from prematurity. Improving neonatal health in Mozambique must address external factors that put newborns at risk of severe infection but also the quality of antenatal, delivery, and postnatal care. Indeed, analysis of SA findings of mothers of neonatal deaths, using data from COMSA, revealed a combination of demand side and health system failures. Most newborn deaths occur within the hours and days following delivery.^36^^,^^37^ In Mozambique, prompt recognition of pregnancy or newborn danger signs followed by formal care seeking, especially for signs of infections, and attention to better planning for quality basic and emergency obstetric and neonatal care will address the continued high rate of neonatal mortality. The SA findings are useful for directing attention to critical actions that the health system must undertake to prevent death. In Niger, this approach has been used along with VA data to inform policy development and maternal, newborn, and child health programming.^38^ The findings in Mozambique are consistent with those observed in Niger, Nigeria, and Malawi, calling for greater attention to danger sign recognition by mothers and prompt careseeking but also the need to improve quality of care for sick newborns, including appropriate referral for emergency care.^39^^–^^41^

Children 1–59 months continue to face high risk of death due to infectious diseases for which simple interventions are available to prevent or treat effectively. Malaria, diarrhea, and lower respiratory infections caused 53% of deaths, and 37% died of other infections. Formal careseeking for child illness is generally high. However, attention must be given to quality of care and referral. The SA analysis found that, for about a quarter of deceased children aged 1–59 months or 5–14 years, no care was sought outside the home for the fatal illness. Furthermore, even though over 70% sought formal care outside, referral for further care was not optimal. This implies that greater attention must be given to addressing child illness treatment by strengthening programs such as integrated management of childhood illness. The 2018 Mozambique malaria indicator survey showed that 69% of under-five children with fever sought care from a health facility, with only 36% on the same day or the day before the fever, whereas only 48% received a malaria diagnostic test.^42^ The findings of the VASA analysis emphasizes the critical need to address child careseeking but most importantly case management for infections such as malaria, diarrhea, and pneumonia. Similar results and implications have been documented in SA studies implemented throughout sub-Saharan African, including in Niger, Nigeria, Tanzania, Cameroon, and Malawi, among others.^43^^,^^44^

The mortality rate among children 5–14 years is the lowest by age group, estimated at 14 per 1,000. The rate is highest in northern and central provinces such as Nampula, Niassa, and Zambezia. Rural areas have twice the rate of urban areas. Injury caused a third of the deaths in this age group, whereas diarrhea, malaria, and pneumonia together caused 37% of deaths, adding to HIV (13%) and other infections (15%).

Estimates of adult mortality among those 15–59 years and 60+ years, are high, reaching 228 and 503 deaths per 1,000. Deaths among people who were 15–59 years old suffered from HIV/AIDS (25%), injury (13%), cancer (10%), and other infections (22%). Among those aged 60+, infections, cancer, and cardiovascular diseases are important causes, accounting for 16% and 15% of deaths, respectively. The COD profile is typical for these age groups, with rising concerns about noncommunicable diseases. However, the deleterious effects of HIV remain acute in Mozambique and require continued prioritization, along with greater attention to noncommunicable diseases such as cancer and stroke. The pathway to survival analysis findings were comparable to those of older children, characterized by less-than-optimal patient care at the facility level. In general, about three-quarters of deaths sought care as a first or second action and reached the first provider alive. However, less than a quarter discharged alive were referred for further care.

The Mozambique COMSA project to establish a sample vital statistics system is the first that integrates VASA data to produce continuous data at national and subnational levels. The link to MITS data to generate more accurate data on causes of child death is unique. The mortality statistics generated can also be used to assess the plausibility of estimates from other sources, such as the population census or the routine health information system. The data produced have been enthusiastically received by the Ministry of Health, and the INS uses the data as the only source for understanding factors related to death and answering programmatic questions related to mortality and COD disparities across the country, including maternal mortality. The data also include additional population-based coverage indicators, such as facility delivery and birth and death registration. COMSA has also been recognized by the Ministry of Health as a strong tool to raise awareness on other sectors regarding the role of different sectors in reducing/eliminating preventable deaths based on the social autopsy data.

The COMSA project in Mozambique is already proving instrumental not only for the usefulness of the wealth of data generated but also for its potential to serve as a sustainable platform for additional targeted disease surveillance, research piloting, and strengthening other systems such as the CRVS, and routine health information. Discussion is ongoing to establish a process linking the COMSA data to the country CRVS, both through the use of these data to validate the completeness report of CRVS and through the transfer of individual events. The COMSA community workers have been trained in selected provinces to assist with the electronic notification of births and deaths into the vital registration system. Using the COMSA platform, a validation exercise of using the COMSA community workers to support registration of births and deaths into the vital registration system by completing the registration forms for events identified within their clusters has been implemented. The preliminary results suggest that the strategy is feasible but will require careful support and training of the community workers for the proper filing of the registration forms. Thus, ensuring complete registration within COMSA clusters and using the community workers to expand the identification and registration of events, even beyond their clusters, are immediate steps that the CRVS will benefit from.

The COMSA data collected were part of the set of background data used by Mozambique in response to the COVID-19. Our analysis of monthly deaths captured by the project did not reveal any signal that would suggest significant excess mortality during the peaks of the pandemic in Mozambique. However, the pandemic has also caused severe disruption in the community data reporting during lockdowns (Supplemental Appendix Figure 3).

To further demonstrate the usefulness of the COMSA-Mozambique platform, a pilot sero-surveillance of multiple diseases using the CDC multiplex beads assays was successfully conducted in one province in early 2020 using the COMSA platform, and data are currently being analyzed. The sero-surveillance was implemented as a proof of concept for building a multi-diseases sero-surveillance based on dried blood spot within the COMSA platform. The pilot was implemented by INS with support from Johns Hopkins University, the CDC, and the London School for Hygiene and Tropical Medicine. In addition to testing for vaccine-preventable diseases and neglected tropical diseases, it included malaria and HIV testing. The pilot presented also a unique opportunity to strengthen the capacity of INS in laboratory equipment and testing of assays. The success of this pilot offers a unique opportunity to extend the approach to other provinces for disease surveillance that the government of Mozambique has prioritized.

The COMSA platform is also being used to test the use of mobile phone interviews for mortality data collection, as an effective strategy for measuring mortality during pandemics such the COVID-19. Another study is currently underway to collect data from households to measure the indirect impact of the COVID-19 on the use of reproductive, maternal, newborn and child health services.

Despite these potentials, the challenges for maintaining the system at a reliable functionality to generate data that are accurate and usable for policy and programs in the country, as well as provide regional and global learning and evidence, should not be underestimated. Like SVRS in other countries, the COMSA data collection architecture relies heavily on community workers receiving modest compensation. These workers are necessary to maintain an active surveillance and contact with the selected communities and continuous monitoring of events. However, data reported by these workers are not usually complete enough and must be complemented with additional retrospective annual data collection. Lags in the follow-up of deaths reported for VASA interviews are frequent, and backlogs of uncompleted VASA interviews can increase rapidly if adequate support is not provided to the VASA data collection. As of September 2021, the project followed only about 70% of deaths reported for successful VASA interviews. The delay was amplified by the COVID-19 pandemic since March 2021. Thus, the COD results presented are affected by a possible distortion in the full sample due to these missing VA data. The human resources required at all levels (i.e., community, province, and central) to maintain smooth administrative, financial, and technical management is paramount to the success of the entire system.

The COMSA platform also faces some methodological limitations. The COMSA clusters were sampled using the 2007 population census master sampling frame. Over time it will require continuous updating of the clusters but also an assessment that the sample remains nationally and provincially representative given population movements and natural crises that the country faces every year. A third of the clusters in the northern province of Cabo-Delgado have become inaccessible due to security concerns. The COMSA sample also excludes very high socio-economic status neighborhoods in large cities because residents in these areas are not receptive to continuous population surveillance based on community worker reporting. The National Institute of Statistics reported often excluding these areas from national surveys given the challenge of reaching these populations. VA data carry greater uncertainties than full or minimally invasive autopsies or medical certification of CODs, and therefore findings must be interpreted cautiously, especially when data are used at the individual level.

COMSA uses a state-of-the-art digital system for data collection that includes mobile phones with ODK data collection at the community level; tablets for VASA data collection; and cloud servers for data storage, analysis, and release. The system allows a near-real time data collection, review, and release and is currently being linked to the DHIS-2 system used for reporting information from health facilities. Discussion is also underway to link the COMSA SRVS to the CRVS. Thus, the COMSA project offers a proof of concept of the feasibility of an SVRS digital system in Africa.

In conclusion, the COMSA platform offers a rich and unique system for mortality and COD determination and monitoring and an opportunity to build a comprehensive surveillance system that addresses multiple health programs. The platform is country owned and run and produces data that are directly relevant for the Ministry of Health and stakeholders for program and policy decision-making. It also represents a country platform on which additional data collection can be built to respond quickly to questions arising from health programs.

## Financial Disclosure

Financial support: This study was funded by the Bill & Melinda Gates Foundation (grant number OPP1163221).

## Supplemental Materials


Supplemental materials
