# Supplementary material for: Countrywide Mortality Surveillance for Action in Mozambique: Results from a National Sample-Based Vital Statistics System for Mortality and Cause of Death
Source: Am J Trop Med Hyg. 2023 Apr 10;108(5 Suppl):5–16. doi: 10.4269/ajtmh.22-0367 (PMC10160865; doi:10.4269/ajtmh.22-0367)
Supplement: Supplementary file 1 [file tpmd220367.SD1.pdf]

SUPPLEMENTAL APPENDIX TABLE 1

Sample size calculation for the COMSA project

Province	U5MR (DHS 2011), reference year 2007	Projected U5MR to 2016*	IMR (DHS 2011), reference year 2007	Projected IMR to 2016*	Relative error margin (2 SE/X) (%)	Absolute error margin (IMR) (2SE)	Average HH size (DHS 2011)	Number of annual births (based on IMR)	Estimated annual under-five deaths	Estimated annual infant deaths	Estimated HHs	Estimated number of SRS EAs (300HH/EAs)	Estimated total population in SRS clusters	Estimated total number of death (based on CDR = 0.010)	Absolute margin of error for U5MR (2 SE)	Estimated number of infant children (based on 4%)	Estimated number of under-five children (based on 18%)
(1)	(2)	(3)	(4)	(5)	(6)	(7)	(8)	(9)	(10)	(11)	(12)	(13)	(14)	(15)	(16)	(17)	(18)
Inhambane	0.058	0.037	0.039	0.026	29	0.007	4.1	2,516	92	64	14,845	49	61,362	614	0.009	2,454	11,045
Nampula	0.067	0.042	0.041	0.027	29	0.008	3.6	2,390	101	64	16,009	53	58,290	583	0.010	2,332	10,492
Maputo City	0.080	0.051	0.061	0.040	25	0.010	4.9	2,132	108	85	10,651	36	52,009	520	0.011	2,080	9,362
Maputo Province	0.096	0.061	0.068	0.045	25	0.011	4.4	1,904	116	85	10,668	36	46,432	464	0.013	1,857	8,358
Niassa	0.101	0.064	0.061	0.040	25	0.010	4.3	2,132	136	85	12,033	40	52,009	520	0.012	2,080	9,362
Sofala	0.105	0.066	0.073	0.048	25	0.012	5.0	1,767	117	85	8,551	29	43,103	431	0.014	1,724	7,759
Gaza	0.110	0.070	0.063	0.041	25	0.010	4.7	2,062	143	85	10,746	36	50,289	503	0.013	2,012	9,052
Manica	0.114	0.072	0.064	0.042	17	0.007	4.4	4,550	328	191	25,405	85	110,977	1,110	0.009	4,439	19,976
Cabo Delgado	0.116	0.073	0.082	0.054	13	0.007	4.1	5,758	422	309	33,933	113	140,439	1,404	0.008	5,618	25,279
Tete	0.129	0.082	0.086	0.056	12	0.007	4.6	6,022	491	339	31,948	106	146,882	1,469	0.008	5,875	26,439
Zambezia	0.142	0.090	0.095	0.062	11	0.007	4.6	6,611	593	412	35,304	118	161,239	1,612	0.008	6,450	29,023
Total								37,844	2,649	1,805	21,0093	700	92,3031	9,230		36,921	16,6146

COMSA = Countrywide Mortality Surveillance for Action; DHS = Demographic and Health Survey; EAs = Enumeration areas; HH = household; IMR = infant mortality rate; SRS = stratified random sample; U5MR = under-five mortality rate.

Assumptions: design effect = 1.3 based on DHS 2011; response rate of 90%; crude birth rate of 41 per 1,000 based on DHS 2011; crude death rate of 10 per 1000 based on 2007 population census. Error margin is 2 × standard error of the estimate.* Projection using annual rate of reduction (2000–2010) based on United Nations Inter-agency Group for Child Mortality Estimation estimates of national infant and under-five mortality rates (2010 was used instead of 2015 because 2015 estimates were based on extrapolation).

SUPPLEMENTAL APPENDIX TABLE 2

Distribution of COMSA population, births deaths, and crude birth and death rates at national level, by place of residence and by province

Population	Total population*		Total births†		Total deaths†		CBR	95% CI of CBR	CDR	95% CI of CDR
	Unweighted	Weighted	Unweighted	Weighted	Unweighted	Weighted				
National	744,732	744,732	44,432	41,880	11,162	10,716	30.2	29.0–31.3	7.9	7.5–8.2
Urban	253,796	223,740	12,555	10,259	3,512	2,917	24.5	23.1–26.0	7.1	6.7–7.6
Rural	490,936	520,992	31,877	31,621	7,650	7,799	32.4	31.1–34.0	8.2	7.7–8.6
Niassa	40,344	50,002	3,545	4,267	651	790	45.5	41.4–50.1	8.6	7.5–9.8
Cabo Delgado	81,187	44,259	5,670	2,951	1,458	779	35.6	32.9–38.7	9.4	8.1–10.6
Nampula	53,879	99,402	3,563	6,169	899	1,545	33.1	30.4–36.0	8.6	7.7–9.6
Zambezia	132,137	157,245	7,764	8,494	2,178	2,439	28.8	25.7–32.1	8.5	7.5–9.5
Tete	112,338	92,298	6,898	5,112	1,231	901	29.6	27.1–32.5	5.4	4.7–6.2
Manica	86,409	51,289	6,159	3,500	1,369	784	36.4	34.5–38.8	8.3	7.6–9.1
Sofala	37,177	56,844	2,503	3,636	626	908	34.1	29.9–38.5	9.0	7.6–10.4
Inhambane	57,408	52,082	2,442	2,142	892	788	21.9	20.0–24.2	7.9	7.0–8.9
Gaza	47,305	57,298	2,315	2,679	742	876	24.9	23.6–26.6	8.2	7.0–9.3
Maputo Province	47,614	46,242	1,649	1,538	473	441	17.7	15.8–19.8	5.1	4.4–5.8
Maputo City	48,934	37,773	1,924	1,392	643	464	19.7	18.4–21.1	6.7	5.9–7.4

CBR = crude birth weight; CDR = crude death rate; COMSA = Countrywide Mortality Surveillance for Action.

* Unweighted population estimated for the year 2020.

† Unweighted population estimated for the period 2019–2020.

Supplemental Appendix Table 3

Mortality rates and 95% CI, COMSA data

	Neonatal rate		Infant mortality rate		Under-five mortality rate		5–14 years old		15–24 years old		25–59 years old		60+ years old	
	Value	95% CI	Value	95% CI	Value	95% CI	Value	95% CI	Value	95% CI	Value	95% CI	Value	95% CI
National	23.2	18.2–28.2	46.0	38.1–53.8	80.0	69.0–90.9	17.7	14.0–21.4	25.6	20.3–30.9	258.3	229.5–287.2	531.2	490.3–572.1
Urban	19.1	10.4–27.8	33.7	22.5–44.9	57.5	40.5–74.5	13.7	8.6–18.7	23.6	16.0–31.3	262.3	219.4–305.1	558.1	504.4–611.8
Rural	24.5	18.6–30.5	50.0	40.3–59.6	87.3	74.1–100.5	19.2	14.4–23.9	26.7	19.7–33.6	256.3	218.4–294.2	520.0	466.5–573.6
Niassa	23.6	3.8–43.3	38.0	14.9–61.0	70.3	33.9–106.7	21.3	2.0–40.6	28.0	11.2–44.8	283.2	195.0–371.4	583.9	413.6–754.2
Cabo Delgado	20.4	8.3–32.4	44.9	28.1–61.7	81.7	58.0–105.4	26.0	13.6–38.3	26.0	11.5–40.5	342.4	209.6–475.1	524.6	397.2–652.0
Nampula	15.1	5.2–25.1	39.5	18.6–60.5	85.2	49.6–120.9	23.8	12.1–35.5	33.2	13.6–52.8	280.5	193.4–367.7	539.9	424.1–655.6
Zambezia	28.3	16.5–40.0	66.3	42.9–89.8	108.6	79.4–137.9	20.1	11.4–28.7	32.9	18.3–47.5	284.5	205.3–363.8	500.8	365.2–636.4
Tete	22.7	7.6–37.9	41.9	24.9–58.8	72.1	47.7–96.5	15.8	5.7–26.0	17.1	7.1–27.2	129.5	77.5–181.4	404.2	291.4–517.0
Manica	30.5	17.8–43.3	54.5	35.9–73.2	84.3	59.5–109.2	14.0	5.8–22.3	27.4	13.3–41.5	278.7	213.8–343.6	501.9	393.8–609.9
Sofola	32.7	6.2–59.2	53.2	20.7–85.7	89.5	48.7–130.3	15.7	0.2–31.3	33.1	4.5–61.8	293.4	185.0–401.8	534.0	417.1–650.8
Inhambane	18.9	6.9–30.9	32.1	14.6–49.7	60.9	29.9–91.9	10.6	1.6–19.7	19.3	6.2–32.3	289.6	190.4–388.7	642.9	515.7–770.0
Gaza	22.2	5.4–39.1	36.1	13.0–59.2	53.7	25.8–81.5	15.0	4.1–26.0	21.2	7.7–34.8	271.4	181.7–361.1	583.7	486.0–681.3
Maputo Prov	14.5	−0.2–29.2	23.5	5.9–41.1	39.1	15.3–62.9	7.5	1.2–13.9	12.8	2.9–22.7	185.0	121.0–249.0	473.2	365.3–581.1
Maputo City	9.9	0.1–19.6	16.7	4.1–29.2	26.9	8.2–45.7	9.3	−2.0–20.6	14.8	2.2–27.4	228.1	173.5–282.7	551.4	435.3–667.6

COMSA = Countrywide Mortality Surveillance for Action.

Supplemental Appendix Table 4

Life expectancy at age *x*, *x* from 0 to 85 years, COMSA data 2019–2020

Age	Mortality rate (ages *x*, *x* + *n*) per 1,000			Probability of death (ages *x*, *x* + *n*) per 1,000			Life expectancy at age *x* (both sexes)		
	Total	Males	Females	Total	Males	Females	Total	Males	Females
0	37.5	39.12	35.91	36.6	38.1	35.1	63.2	60.4	66.1
1	7.6	8.39	6.90	29.9	32.8	27.1	64.6	61.8	67.5
5	2.0	2.24	1.71	9.8	11.1	8.5	62.5	59.8	65.3
10	1.4	1.55	1.25	7.0	7.7	6.2	58.1	55.5	60.9
15	2.1	1.90	2.24	10.3	9.4	11.1	53.5	50.9	56.2
20	3.2	3.61	2.79	15.8	17.9	13.8	49.0	46.3	51.8
25	5.4	5.65	5.07	26.4	27.9	25.0	44.8	42.1	47.5
30	7.1	7.62	6.61	34.8	37.4	32.5	40.9	38.3	43.7
35	8.6	10.09	7.37	42.3	49.2	36.2	37.3	34.7	40.0
40	9.9	12.35	7.63	48.3	59.9	37.4	33.9	31.3	36.5
45	10.2	11.88	8.56	49.6	57.7	41.9	30.5	28.2	32.8
50	13.9	16.37	11.69	67.4	78.6	56.8	26.9	24.7	29.1
55	16.7	19.80	13.79	80.0	94.3	66.6	23.7	21.6	25.7
60	22.0	24.82	19.56	104.2	116.8	93.2	20.5	18.6	22.4
65	29.7	34.59	25.65	138.0	159.2	120.5	17.6	15.7	19.4
70	37.2	44.10	31.69	170.4	198.6	146.8	15.0	13.2	16.7
75	53.9	63.71	46.19	237.7	274.8	207.0	12.6	10.9	14.2
80	66.3	84.84	54.74	284.5	350.0	240.7	10.8	9.1	12.2
85	110.6	130.75	97.22	1,000.0	1,000.0	1,000.0	9.0	7.6	10.3

COMSA = Countrywide Mortality Surveillance for Action.

Supplemental Appendix Table 5

Cause specific mortality fractions by age group at national, regional, and sex levels

Causes of death	National*			Central			Northern			Southern			Females			Males		
	Number of deaths (unweighted)	%	95% CI	Number of deaths (unweighted)	%	95% CI	Number of deaths (unweighted)	%	95% CI	Number of deaths (unweighted)	%	95% CI	Number of deaths (unweighted)	%	95% CI	Number of deaths (unweighted)	%	95% CI
Neonatal	547			254			230			62			236			311		
Prematurity	55	10.0	6.8–13.2	70	28	22.7–34.1	51	23	17.4–28.4	17	28	16.2–38.8	65	27	20.8–32.2	73	25	20.3–30.1
IPRE	109	20.0	14.2–26.4)	79	29	23.5–34.9	55	22	16.7–27.7	25	42	29.5–54.5	60	22	17.0–27.8	99	32	26.2–36.8
Congenital malformation	11	2.0	0.5–5.1	0	0	0–0	1	0	0–0	0	0	0–0	1	0	–0.4–1.2	0	0	0–0
Infection	339	62.0	55.9–68.6	95	38	32.3–44.5	118	53	46.4–59.6	17	25	14.0–36.0	103	48	41.6–54.6	128	40	34.4–45.6
Other	27	5.0	2.2–9.4	10	4	1.5–6.5	5	2	–0.1–3.1	3	5	–0.3–11.1	7	3	0.5–4.7	11	3	1.3–5.3
Child 1–59 months	1,194			480			616			98			568			626		
Diarrhea	251	21.0	15.7–27.0	112	24	20.0–27.8	153	26	22.7–29.7	10	9	3.3–14.9	127	24.2	20.6–27.8	148	23.8	20.4–27.2
Malaria	263	22.0	15.3–28.9	105	22	18.6–26.2	115	18	14.5–20.7	14	15	7.8–22.2	113	18.4	15.1–21.7	121	19.3	16.1–22.5
Lower respiratory infection	107	9.0	5.4–12.8	70	14	11.2–17.6	87	13	10.0–15.4	25	29	19.7–38.1	90	14.5	11.5–17.5	92	14.8	12.0–17.6
HIV	12	1.0	0.3–2.1	9	2	0.8–3.4	26	4	2.4–5.6	6	6	1.2–10.8	22	3.5	2.0–5.0	19	3.6	2.1–5.1
Other infections	442	37.0	26.8–47.8	103	20	16.5–23.9	156	26	22.2–29.2	14	14	7.1–21.3	140	26	22.3–29.7	133	20.2	17.0–23.4
Severe malnutrition	48	4.0	1.5–7.5	21	4	2.4–6.2	26	4	2.5–5.7	5	4	0.2–8.4	19	2.9	1.5–4.3	33	5.4	3.6–7.2
Other	72	6.0	2.8–9.8	60	13	9.6–15.6	53	10	7.4–12.2	24	22	14.0–30.8	57	10.5	7.9–13.1	80	13	10.3–15.7
Child 5–14 years old	310			112			152			46			135			175		
Diarrhea	33	10.5	7.1–13.9	11	13	6.4–18.6	21	12.4	7.2–17.6	0	0	0–0	13	7.8	3.3–12.3	19	12.6	7.7–17.5
Malaria	39	12.6	8.9–16.3	20	13	6.9–19.5	22	12.9	7.6–18.2	4	10	1.3–18.7	19	12.9	7.2–18.6	27	12.3	7.4–17.2
Lower respiratory infection	13	4.3	2.-–6.6	4	2	–0.5–5.1	5	3.1	0.3–5.9	6	11.9	2.5–21.3	5	2.7	0–5.4	10	5.5	2.1–8.9
HIV	18	5.7	3.1–8.3	1	1	–0.8–3.0	7	6.1	2.3–9.9	6	12.8	3.1–22.5	5	5.7	1.8–9.6	9	5.6	2.2–9.0
Other infection	98	31.7	26.5–36.9	31	34	25.4–43.0	48	32.5	25.1–39.9	10	24.2	11.8–36.6	45	32.2	24.3–40.1	44	31.3	24.4–38.2
Injury	61	19.7	15.3–24.1	26	25	16.6–32.6	21	14.5	8.9–20.1	14	28.2	15.2–41.2	25	19.8	13.1–26.5	36	19.6	13.7–25.5
Tuberculosis	4	1.2	0–2.4	0	0	0–0	2	2.2	–0.1–4.5	0	0	0–0	0	0	0–0	2	2.1	0–4.2
Other	45	14.5	10.6–18.4	19	12	6.1–18.1	26	16.2	10.3–22.1	6	12.8	3.1–22.5	23	18.9	12.3–25.5	28	11	6.4–15.6
Age 15–59	2,169			629			589			589			1,010			1,156		
HIV	547	25.2	23.3–27.1	135	26	23.4–29.2	153	26	22.2–29.4	153	26	22.2–29.4	300	29	25.7–31.3	252	22.2	19.8–24.6
Cancer	223	10.3	9.0–11.6	47	12	9.8–14	56	10	7.3–12.3	56	10	7.3–12.3	113	12	9.8–13.8	96	9	7.3–10.7
Injury	286	13.2	11.7–14.7	101	9	7.1–10.7	108	18	15.0–21.4	108	18	15.0–21.4	80	9	7.0–10.6	215	17.1	14.9–19.3
Maternal	124	5.7	4.7–6.7	42	6	4.7–7.9	23	4	2.5–5.7	23	4	2.5–5.7	126	12	10.2–14.4	0	0	0–0
Cardiovascular diseases	113	5.2	4.2–6.2	32	5	3.7–6.5	33	5	3.5–7.1	33	5	3.5–7.1	46	4	3.1–5.7	64	5.9	4.5–7.3
Tuberculosis	82	3.8	3.0–4.6	28	4	2.6–5.2	22	4	2.1–5.3	22	4	2.1–5.3	40	4	2.6–5.0	45	3.8	2.7–4.9
Other infections	482	22.2	20.4–24.0	149	24	20.9–26.5	105	18	14.5–20.7	105	18	14.5–20.7	193	20	17.0–22.0	277	24.5	22.0–27.0
Other	310	14.3	12.8–15.8	95	14	11.5–15.9	89	15	12.4–18.4	89	15	12.4–18.4	112	11	9.0–13.0	207	17.3	15.1–19.5
Age 60+	1,710			464			554			689			885			822		
HIV	103	6	4.9–7.1	21	4	1.9–6.1	37	7	4.9–8.7	43	6	4.4–8.2	56	6	4.8–8.0	45	5.5	3.9–7.1
Cancer	272	15.9	14.1–17.7	67	15	11.3–19.1	108	19	15.7–21.7	99	14	11.3–16.5	143	16	13.8–18.8	131	15.4	12.9–17.9
Cardiovascular diseases	258	15.1	13.4–16.8	62	14	9.9–17.3	61	12	9.5–14.5	123	18	15.4–21.2	133	16	13.4–18.4	113	14.1	11.7–16.5
Injury	123	7.2	5.9–8.5	30	6	3.5–8.7	37	6	4.2–7.8	63	9	6.5–10.7	63	7	5.1–8.5	67	7.6	5.8–9.4
Tuberculosis	104	6.1	4.9–7.3	33	7	4.6–10.2	37	7	5.2–9.2	32	5	3.0–6.2	35	4	2.6–5.2	67	8.5	6.6–10.4
Other infections	407	23.8	21.7–25.9	130	28	23.2–32.8	146	27	23.6–30.4	132	19	16.0–22.0	200	22	19.4–25.0	208	25.5	22.5–28.5
Other	443	25	23.8–	121	2	20.5–	128	22	19.2–	197	29	25.5–	255	28	25.3–	191	23	20.3–

		.9	28.0		5	29.9			25.6			32.5			31.3		.2	26.1

IPRE = intrapartum-related event.

* Results for neonates and children 1–59 months are calibrated.

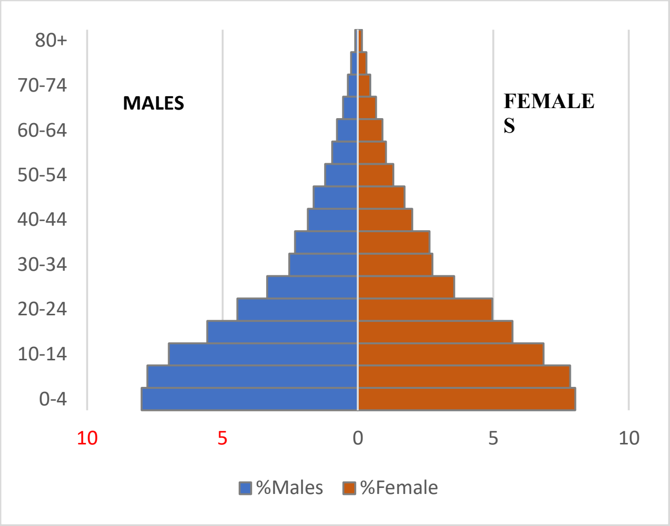

Supplemental Appendix Figure 1. Age and sex pyramid, COMSA Mozambique.

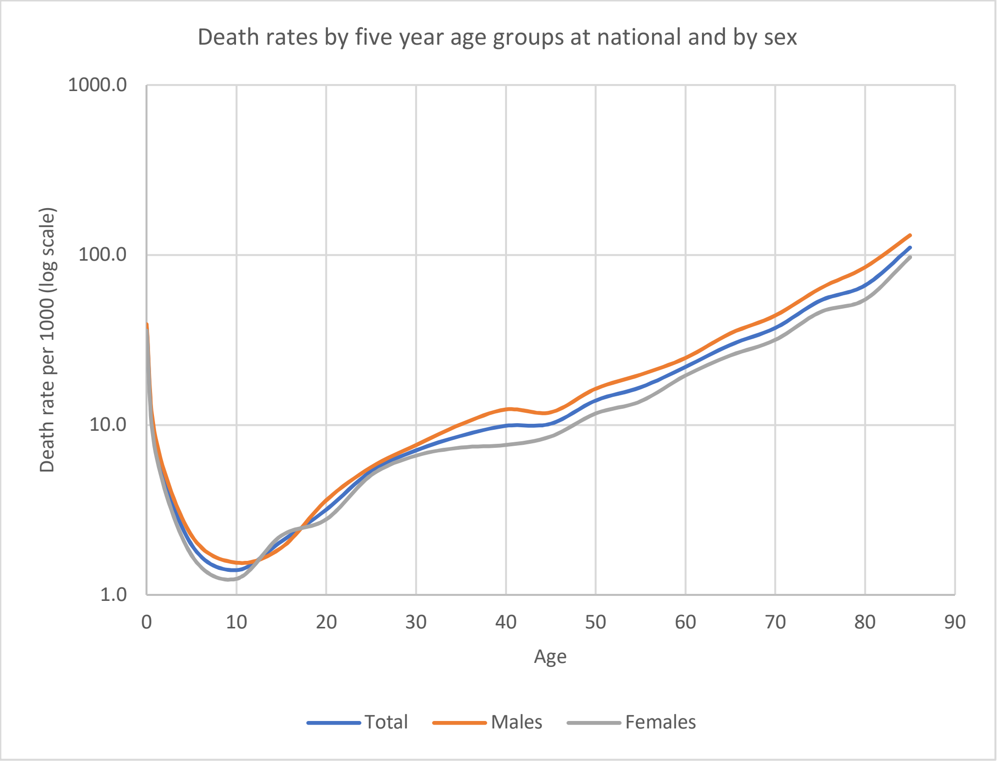

SUPPLEMENTAL APPENDIX FIGURE 2. Distribution of death rates by age at national level and by sex.

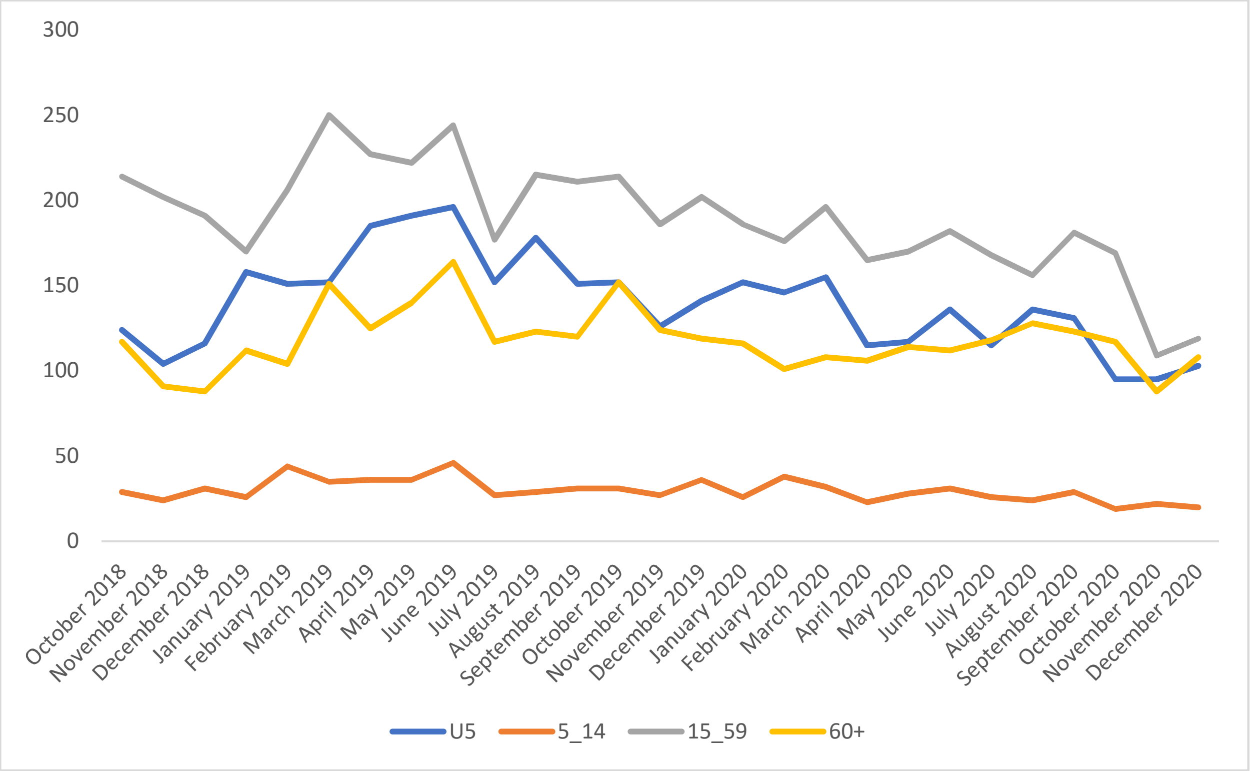

Supplemental Appendix Figure 3. Monthly number of deaths by age group between October 2018 and December 2020
